# The Hidden Impact of Cyberbullying on Teens’ Orthodontic Decisions: A Mixed-Methods Study

**DOI:** 10.4317/jced.63402

**Published:** 2025-11-30

**Authors:** Siddharth Sonwane, Shweta Sonwane, Purvi Awasthi

**Affiliations:** 1 Professor and Head. Department of orthodontics Mansarovar Dental College Bhopal, India; 2 Associate Professor. Department of oral surgery. Government Dental College Nagpur, India; 3 Post-Graduate student. Department of orthodontics. Mansarovar Dental College Bhopal, India

## Abstract

**Background:**

Cyberbullying, particularly appearance-related harassment, is a growing psychosocial concern among adolescents. Dentofacial features, often targeted in online ridicule, may influence adolescents' health behaviors, including acceptance of orthodontic treatment. Objective: This study examined the association between cyberbullying victimization and orthodontic treatment acceptance among adolescents using a mixed-methods approach.

**Material and Methods:**

A convergent parallel mixed-methods design was employed among 200 adolescents aged 12-18 years from schools and orthodontic clinics. Cyberbullying exposure was assessed using the Cyberbullying Victimization Scale, and orthodontic treatment acceptance was measured using a modified 8-item scale. Quantitative analysis included Pearson's correlation and multivariate regression. In-depth interviews with 20 participants were thematically analyzed to explore underlying motivations.

**Results:**

A total of 38.5% of adolescents reported moderate to high cyberbullying exposure, with 23% experiencing appearance-related insults. Adolescents exposed to cyberbullying demonstrated significantly higher orthodontic treatment acceptance scores (31.2 ± 4.3) compared with non-victimized peers (27.6 ± 4.8; p &lt; 0.001). Cyberbullying remained an independent predictor of treatment acceptance ( = 0.39, p &lt; 0.001), explaining 31% of the variance. Qualitative themes revealed that adolescents perceived orthodontics as a strategy to counteract ridicule ("Seeking Change to Escape Bullying") and that online commentary strongly shaped self-perception ("Social Media Mirrors Self-Perception").

**Conclusions:**

Appearance-based cyberbullying significantly influences adolescents' motivation to pursue orthodontic treatment. These findings highlight the need for orthodontists to address psychosocial factors in clinical decision-making and to integrate supportive counseling within treatment planning.

**Clinical Significance:**

• Adolescents exposed to appearance-related cyberbullying are more likely to seek orthodontic care, emphasizing the role of psychosocial influences in treatment demand. • Orthodontists should consider screening for cyberbullying experiences during consultations to better understand patient motivations. • Integrating psychological support alongside orthodontic treatment may improve overall well-being and treatment satisfaction.

## Introduction

Adolescence is a pivotal stage for personal identity, where visible dentofacial irregularities often lead to social marginalization-particularly in settings that enforce strict appearance norms (1 , 2). Malocclusion, especially in the anterior region, can alter facial aesthetics and frequently becomes a target for teasing or peer ridicule (3). The rise of social media has dramatically expanded these experiences, shifting them from physical settings into the digital realm (4). Cyberbullying-defined as intentional, repeated harm carried out via electronic communication-has emerged as a significant psychosocial stressor among adolescents, notably in the form of appearance-related harassment (5 , 6). Victims commonly display decreased self-esteem, heightened anxiety or depressive symptoms, body dissatisfaction, psychosomatic complaints such as headaches or sleep disturbances, and increased social withdrawal (7). These psychological outcomes may critically influence their health-seeking behaviors and decision-making. Although traditional bullying has been associated with heightened acceptance and pursuit of orthodontic treatment, the impact of online aggression-especially targeting dental or facial features-remains understudied (8 - 10). Orthodontic intervention during adolescence is shaped by a complex interplay of esthetic concern, peer expectations, and psychosocial wellbeing (11). Adolescents who experience negative feedback about their dental appearance often view orthodontic treatment as more necessary and are more open to pursuing it (12). Yet, there is limited research exploring whether cyberbullying acts as a motivator for seeking such treatment. Given the pervasive digital presence of today's adolescents and the profound psychosocial repercussions of cyberbullying, it is crucial to examine how such online experiences influence attitudes toward orthodontic care. This study thus utilizes a mixed-methods design-integrating quantitative assessment with qualitative interviews-to explore the relationship between cyberbullying victimization and orthodontic treatment acceptance, while illuminating the underlying motivations and perceptions.

## Material and Methods

- Study Design A convergent parallel mixed-methods design was employed to comprehensively examine the relationship between cyberbullying and orthodontic treatment acceptance among adolescents. This approach permitted simultaneous collection and integration of quantitative and qualitative data, blending statistical patterns with participants' lived experiences (11). - Setting and Duration The study was conducted across selected secondary schools and orthodontic clinics in Bhopal, central India, from October 2024 to April 2025. - Ethical Approval Ethical approval was secured from the Institutional Ethical Committee of Mansarovar Dental College, Bhopal, Madhya Pradesh, India (Ref: IEC-56/2024-5/77). Written informed consent was obtained from all participants and their legal guardians. The study adhered to the principles of the Declaration of Helsinki (13). - Study Population and Sampling Adolescents aged 12-18 years who used social media regularly (30 minutes/day) and had no prior orthodontic treatment were eligible. Individuals with cognitive impairments or psychiatric illnesses that could impede participation were excluded. For the quantitative component, a sample size of 200 was estimated using G*Power version 3.1, assuming a moderate effect size (r = 0.3), = 0.05, and power (1-) = 0.90 (13). Stratified random sampling across schools and clinics ensured representative distribution by age and gender. For the qualitative component, 20 participants were purposively selected from the quantitative sample to maximize diversity in age, gender, extent of cyberbullying exposure, and treatment readiness. - Data Collection Tools Quantitative Instruments Cyberbullying Victimization Scale: A 10-item scale adapted from the Cyberbullying and Online Aggression Survey by Hinduja and Patchin assessed the frequency of cybervictimization on a 5-point Likert scale (0 = Never to 4 = Daily); higher scores indicated greater exposure. Orthodontic Treatment Acceptance Questionnaire: A modified 8-item tool evaluated willingness to undergo orthodontic treatment, perceived social norms, and perceived necessity, using a 5-point Likert scale (1 = Strongly disagree to 5 = Strongly agree). Higher cumulative scores reflected greater treatment acceptance. Self-Esteem Screening: A 4-item short form of the Rosenberg Self-Esteem Scale was used to control for baseline confidence levels. Demographics &amp; Social Media Use: Participants reported age, gender, average daily social media use, and smartphone ownership. Qualitative Interviews A semi-structured interview guide explored appearance-related online harassment, emotional responses, and motivations for pursuing orthodontic correction. Interviews (20-30 minutes) were conducted in English, audio-recorded with consent, and transcribed verbatim. - Data Analysis Quantitative Analysis Statistical analysis was conducted using SPSS v26.0 (IBM Corp., Armonk, NY, USA). Descriptive statistics summarized participant characteristics. Pearson's correlation assessed the relationship between cyberbullying and treatment acceptance. Group comparisons (e.g., high vs. low bullying exposure) utilized independent t-tests or ANOVA. Multiple linear regression adjusted for self-esteem, gender, and social media use. QualitativeAnalysis Data were analyzed using reflexive thematic analysis following Braun and Clarke's six-phase framework (13). Transcripts were coded inductively in NVivo (QSR International) by two independent researchers, with discrepancies resolved via discussion. Data Integration Quantitative and qualitative findings were integrated during interpretation to enable triangulation and enhance study credibility. Convergences and divergences across data types were identified to develop a holistic understanding of how cyberbullying affects orthodontic treatment acceptance.

## Results

- Participant Characteristics A total of 200 adolescents were included in the quantitative analysis (mean age: 15.6 ± 1.9 years; 54% female, 46% male). The majority (87%) reported daily social media use of 1 hour, and 72% owned a personal smartphone (Table 1).

Table 1

**Figure 1:**
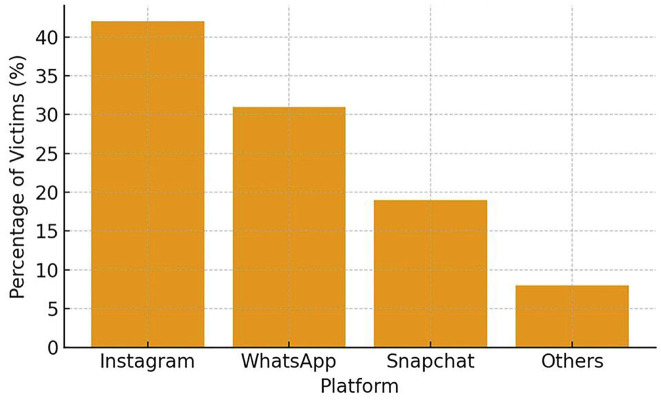
Platforms where cyberbullying occurred.

Participants were evenly distributed across early (12-14 years), mid (15-16 years), and late adolescence (17-18 years). - Cyberbullying Prevalence and Exposure Overall, 38.5% (n = 77) of adolescents reported moderate to high levels of cyberbullying, with 23% (n = 46) specifically experiencing appearance-related victimization. Among the platforms reported, Instagram accounted for the highest proportion of incidents (42%), followed by WhatsApp (31%) and Snapchat (19%) (Table 2, Fig. 1).

Table 2 1

**Figure 2:**
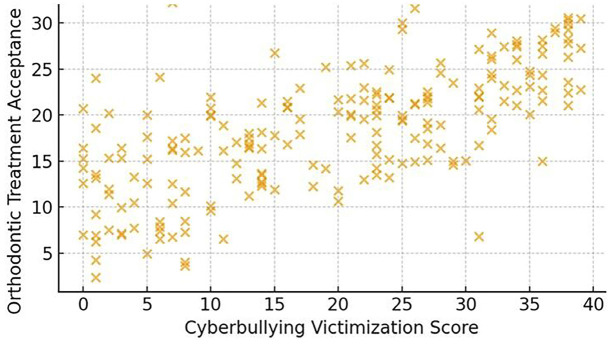
Cyberbullying and orthodontic treatment acceptance.

- Orthodontic Treatment Acceptance The mean orthodontic treatment acceptance score was 29.3 ± 5.1 (maximum score = 40). Adolescents exposed to cyberbullying had significantly higher acceptance scores (31.2 ± 4.3) compared with their non-victimized peers (27.6 ± 4.8; t = 4.93, p &lt; 0.001) (Table 3, Fig. 2).

Table 3

2

**Figure 3:**
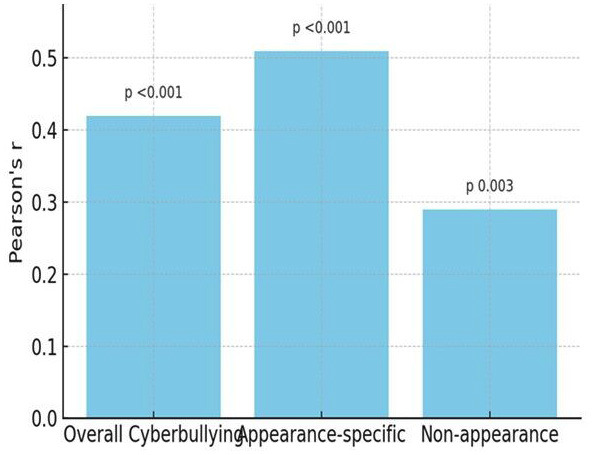
Correlation between cyberbullying and treatment acceptance.

- Correlation Analysis Pearson's correlation demonstrated a moderate positive association between overall cyberbullying and orthodontic treatment acceptance (r = 0.42, p &lt; 0.001). Appearance-specific cyberbullying was more strongly correlated (r = 0.51, p &lt; 0.001), whereas non-appearance-related bullying showed a weaker but significant relationship (r = 0.29, p = 0.003) (Table 4, Fig. 3).

Table 4

3

**Figure 4:**
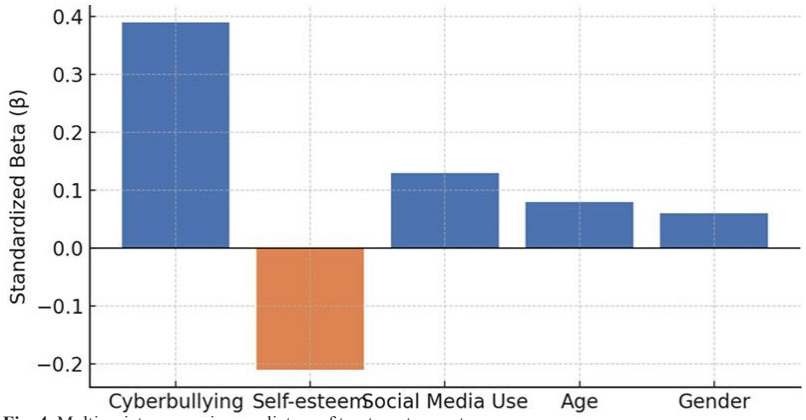
Multivariate regression predictors of treatment acceptance.

- Multivariate Regression Analysis After adjusting for gender, age, social media use, and self-esteem, cyberbullying victimization remained a significant independent predictor of orthodontic treatment acceptance ( = 0.39, p &lt; 0.001). Lower self-esteem ( = -0.21, p = 0.004) and higher daily social media use ( = 0.13, p = 0.045) also significantly predicted treatment acceptance. Age and gender were not significant predictors. The final model explained 31% of the variance in treatment acceptance (F(5, 194) = 18.21, R² = 0.31, p &lt; 0.001) (Table 5, Fig. 4).

Table 5

4

- Qualitative Themes Thematic analysis of 20 interviews revealed three central themes: 1. "Being Judged Online" - Adolescents described feeling humiliated and embarrassed by derogatory comments, memes, or posts targeting their dental appearance. 2. "Seeking Change to Escape Bullying" - Victims expressed that pursuing orthodontic treatment was motivated by the desire to reduce online ridicule and achieve greater social acceptance. 3. "Social Media Mirrors Self-Perception" - Adolescents reported internalizing digital feedback, with social validation strongly influencing their self-concept and health behavior decisions.

## Discussion

This mixed-methods study investigated the influence of cyberbullying-particularly appearance-related forms-on adolescents' acceptance of orthodontic treatment. The findings demonstrated that adolescents exposed to online harassment, especially insults directed at dental or facial features, exhibited a significantly higher willingness to accept orthodontic treatment compared with their non-victimized peers. These results highlight the growing psychosocial role of digital environments in shaping adolescent health behaviors. - Cyberbullying Prevalence and Appearance-Related Harassment In this study, 38.5% of participants reported moderate to high levels of cyberbullying, with nearly one-quarter experiencing appearance-related insults. This prevalence aligns with recent reports showing that visual identity, especially dentofacial features, is a frequent target of online aggression (1 , 2). Platforms such as Instagram and Snapchat-where image-sharing is central-were among the most common sites of victimization, supporting prior evidence that adolescents are vulnerable to harassment in image-centric digital spaces (3). Classic research has long recognized dentofacial appearance as central to peer perception and social acceptance (4 , 5), and in today's digital context, these vulnerabilities appear magnified. - Cyberbullying and Orthodontic Treatment Acceptance Adolescents who experienced appearance-based cyberbullying scored significantly higher on orthodontic treatment acceptance scales. Pearson correlation and regression analyses confirmed cyberbullying as an independent predictor of treatment motivation, even after controlling for confounders. These findings resonate with earlier studies reporting that negative self-image, peer ridicule, and social competitiveness strongly influence orthodontic demand (6 - 8). Notably, the current results underscore that adolescents' treatment-seeking extends beyond clinical malocclusion severity, reflecting a broader psychosocial model of healthcare decision-making. Conversely, some studies have argued that treatment motivation is largely driven by clinical factors rather than psychosocial influences (9 , 10). These discrepancies may reflect differences in study populations and time periods; older studies were conducted before the widespread influence of social media. Today's adolescents navigate a digital culture in which self-presentation and online validation are integral to identity formation, thereby amplifying the role of psychosocial pressures in treatment decisions. - Insights from Qualitative Findings Thematic analysis enriched the quantitative results by contextualizing adolescents' lived experiences. Themes such as "Being Judged Online" and "Seeking Change to Escape Bullying" highlighted how negative digital interactions directly motivated participants to pursue orthodontic correction. The theme "Social Media Mirrors Self-Perception" further emphasized how adolescents internalize online commentary, with digital validation shaping health-related decisions. These findings mirror prior qualitative work underscoring the emotional toll of aesthetic nonconformity and the role of social approval in shaping treatment uptake (11). - Strengths and Limitations A major strength of this study lies in its convergent parallel mixed-methods design, which allowed for triangulation of quantitative associations with qualitative narratives. The use of validated instruments for both cyberbullying and treatment acceptance enhanced reliability, while regression analysis identified cyberbullying as an independent predictor. However, limitations must be acknowledged. The cross-sectional design precludes causal inference. Reliance on self-reported measures introduces the possibility of recall and social desirability bias. The study sample, drawn primarily from urban schools and clinics, may not fully represent adolescents from rural or socioeconomically diverse backgrounds, thereby limiting generalizability. - Future Directions Future research should employ longitudinal designs to explore causal pathways between cyberbullying and orthodontic decision-making. Investigating the moderating roles of parental support, school-based interventions, and peer networks may provide a more comprehensive understanding. Additionally, integrating psychosocial screening into orthodontic consultations could help clinicians identify adolescents seeking treatment due to appearance-related distress. Collaboration between orthodontists, psychologists, and educators may enhance holistic care for vulnerable adolescents.

## Conclusions

This study establishes that appearance-based cyberbullying significantly increases adolescents' acceptance of orthodontic treatment. As digital platforms increasingly shape peer interactions and self-perception, orthodontists should remain attentive to the psychosocial motivations underlying treatment requests. Incorporating psychological support and counseling into orthodontic practice may better address the holistic needs of adolescents, particularly those whose treatment-seeking behavior is influenced by online victimization.

## Figures and Tables

**Table 1:** Table Demographic and Social Media Use Characteristics (n = 200).

Variable	n (%) / Mean ± SD
Age (years)	15.6 ± 1.9
Gender	
Male	92 (46%)
Female	108 (54%)
Daily Social Media Use	
< 1 hour	26 (13%)
≥ 1 hour	174 (87%)
Smartphone Ownership	144 (72%)

1

**Table 2:** Table Prevalence of Cyberbullying and Platform Distribution.

Cyberbullying Metric	n (%)
Any Cyberbullying (moderate to high)	77 (38.5%)
Appearance-related Cyberbullying	46 (23%)
Most Common Platforms	
Instagram	84 (42%)
WhatsApp	62 (31%)
Snapchat	38 (19%)

2

**Table 3:** Table Orthodontic Treatment Acceptance Score Comparison.

Group	Mean ± SD	t / F	p-value
Cyberbullied (any form)	31.2 ± 4.3	t = 4.93	< 0.001
Not Cyberbullied	27.6 ± 4.8		

3

**Table 4:** Table Pearson Correlation between Variables.

Variable Correlation	r-value	p-value
Cyberbullying & Treatment Acceptance	0.42	< 0.001
Appearance-specific Bullying & Acceptance	0.51	< 0.001
Non-appearance Cyberbullying & Acceptance	0.29	0.003

4

**Table 5:** Table Multivariate Regression Analysis Predicting Orthodontic Treatment Acceptance.

Predictor Variable	Î² (Standardized Coefficient)	p-value
Cyberbullying Victimization	0.39	< 0.001
Self-esteem	-0.21	0.004
Daily Social Media Use	0.13	0.045
Age	0.08	0.210
Gender	0.06	0.318

5

## Data Availability

The datasets used and/or analyzed during the current study are available from the corresponding author.

## Notes

Use of AI: During the preparation of this manuscript, the authors used ChatGPT (OpenAI, GPT-4, June 2024 version) for support in refining text structure, grammar, and formatting of academic sections. The authors have thoroughly reviewed and edited the output and take full responsibility for the content of this publication.
